# Mets-IR as a predictor of cardiovascular events in the middle-aged and elderly population and mediator role of blood lipids

**DOI:** 10.3389/fendo.2023.1224967

**Published:** 2023-07-17

**Authors:** Taoming Qian, Xiaomeng Sheng, Pengyu Shen, Yuting Fang, Yao Deng, Guoliang Zou

**Affiliations:** ^1^ Graduate School, Heilongjiang University of Chinese Medicine, Harbin, Heilongjiang, China; ^2^ Department of Oncology, Second Ward, The First People’s Hospital of Bei’an City, Harbin, Heilongjiang, China; ^3^ Cardiovascular Disease Branch One, First Affiliated Hospital of Heilongjiang University of Chinese Medicine, Harbin, Heilongjiang, China

**Keywords:** CHARLS, METS-IR, cardiovascular disease, stroke, heart disease

## Abstract

**Background:**

Cardiovascular disease (CVD) is a global health concern, with a significant impact on morbidity and mortality rates. Using fasting glucose, fasting triglycerides, body mass index (BMI), and high-density lipoprotein cholesterol (HDL-C), the metabolic score of insulin resistance (Mets-IR), a novel index created by Mexican researchers to assess insulin sensitivity, is a more precise way to measure insulin sensitivity. This study aimes to explore the association between Mets-IR and CVD, as well as investigate the potential mediating role of of low-density lipoprotein cholesterol (LDL-C).

**Methods:**

The study’s data came from the 2011 and 2018 China Health and Retirement Longitudinal Studies (CHARLS). We used three logistic regression models to account for the potential effects of ten factors on cardiovascular disease/stroke/heart disease. Moreover, We performed mediation analyses to evaluate the role of LDL-C in the association between Mets-IR and incident CVD.

**Results:**

This study comprised 4,540 participants, of whom 494 (10.88%) were found to develop disease (CVD). Each interquartile range (IQR) increased in Mets-IR raised the risk of developing CVD by 38% (OR=1.38; 95% CI, 1.21-1.56) and there was a linear dose-response relationship between Mets-IR and the risk of new-onset cardiovascular disease, stroke, and heart disease (*P*_overall_<0.05, *P*_non-linear_>0.05). Approximately 5% (indirect effect/total effect) of the significant association of Mets-IR with stroke was mediated by LDL-C, respectively. With the addition of Mets-IR to the base model, the continuous net reclassification improvement and integrated discrimination improvement for predicting cardiovascular disease increased by 0.175 (P <0.001) and 0.006 (*P <*0.001), respectively.

**Conclusion:**

ets-IR is associated with an increased risk of cardiovascular disease/stroke/cardiac issues, with LDL-C mediating these relationships. Improving insulin sensitivity and lipid regulation may be essential and effective preventive measures for cardiovascular events.

## Introduction

Cardiovascular disease (CVD) now has the greatest rates of morbidity as well as mortality around the world. Approximately one-third of all deaths worldwide each year are attributable to CVD. Ischemic heart disease and stroke, two cardiovascular conditions that are the main killers, are both clinical symptoms of atherosclerosis. They are responsible for 84.9% of CVD deaths (1). As the population ages, the worldwide burden of CVD will continue to increase, particularly in middle-aged as well as older adults (2). With rising urbanization and lifestyle changes, the increasing burden of CVD in China has emerged as an important public health issue currently. The number of people who died from CVD each year in 1990 was 2.51 million. However, in 2016, the number had grown to 3.97 million. CVD not only affects the physical and mental health of patients and is closely associated with adverse emotions such as anxiety and depression (3, 4). Moreover, it is also a major source of financial burden and distress (5), which will lead to a greater medication burden for older adults with CVD as they usually have other chronic conditions in combination (6).

Insulin resistance (IR) plays a crucial role in the development of vascular dysfunction. It is closely connected with the onset of vascular calcification, coronary atherosclerosis, and cardiovascular disease (7). IR cause disorders of glucolipid metabolism, increased production of glycosylated products and deposition around blood vessels, resulting in decreased vascular elasticity and increased fibrosis (8). It also interferes with insulin signaling through endothelial cells, impairing insulin-stimulated glucose metabolism and accelerating atherosclerosis. In addition, insulin resistance is associated with an inflammatory response, inducing the secretion of inflammatory mediators by a variety of inflammatory cells, which in turn affects vascular function (9). It has been suggested that adipose tissue IR is one of the significant risk factors for aortic valve calcification and predicts cardiovascular outcomes (10). A study from Japan found that higher HOMA-IR levels were independently linked to the development and progression of coronary artery calcification (CAC) (11).

The Glucose clamp technique is the accepted gold standard for determining insulin sensitivity in humans (12). Nevertheless, it is invasive and expensive, and its clinical application is limited. Researchers from Mexico created the innovative tool called the metabolic score for insulin resistance (Mets-IR) to assess insulin sensitivity (13), consisting of fasting glucose, fasting triglycerides, body mass index (BMI), and high-density lipoprotein cholesterol (HDL-C), and it is a more accurate indicator of insulin sensitivity. Previous papers have confirmed that Mets-IR is linked with a rising risk of developing metabolic diseases such as T2DM, hyperuricemia, and hypertension (14, 15). A cross-sectional study with 802 subjects explored the connection between Mets-IR and coronary heart disease (CAD) (16), which found significant predictors of Mets-IR and CAD. However, due to its limited sample size and cross-sectional design, the study was unable to clarify the causal link between Mets-IR levels and CAD risk.

As a result, the purpose of this study was to build a prospective cohort study based on CHARLS, the Chinese Longitudinal Study of Health and Retirement. We looked into the link between Mets-IR and CVD/Stroke/Heart disease. The role of LDL-C as a mediator in the preceding relationship is also investigated.

## Method

### Study population

The National Development Institute of Peking University conducted the CHARLS survey in 2011 and 2018; the results can be seen at http://charls.pku.edu.cn. The database gathers a variety of high-quality microdata that reflect families and individuals of middle-aged and elderly Chinese citizens who are 45 years of age and older in order to study the country’s population aging issue and advance multidisciplinary aging research. In 2011, 150 county-level units, 450 village-level units, and over 17,000 people in roughly 10,000 households were included in the CHARLS China Baseline Survey. Every two to three years, these samples are examined.

In 2011 and 2018, professionals took venous blood samples from participants who had not eaten or drunk anything for at least 12 hours. Complete blood cell counts were then carried out on-site. Other samples were transported to Beijing Central Laboratory (You ‘anmen Clinical Laboratory Center, Capital Medical University) for additional testing while whole blood samples were kept at 4°C. Enzyme colorimetry was used to measure the levels of glucose, triglycerides (TG), low-density lipoprotein cholesterol (LDL-C), and high-density lipoprotein cholesterol (HDL-C). Professionals used physical examination equipment to measure the pertinent body mass index (BMI) statistics for height and weight.

The Ethical Review Committee at Peking University gave preliminary permission to CHARLS in 2008 (IRB00001052-11,015). The methodology complied with all relevant CHARLS criteria and recommendations. Each volunteer completed an informed consent form before agreeing to take part in CHARLS.

In the end, we came up with the following inclusion standards for the study’s participants: 45 years of age or older; full demographic information, including gender, age, education level, marital status, and place of residence; full behavioral health information, including drinking and smoking patterns and sleeping time; full relevant laboratory examination information, including blood glucose and lipids; full relevant past medical history, including developed heart disease and developed stroke; and full relevant physical examination information. The 4540 eligible participants who met these inclusion criteria were all included.

## Measurement

### Assessment of Mets-IR

**BMI:** body mass (kg)/the square of height (m^2^)

**TG/HDL-C** = TG (mg/dL) ÷ HDL-C (mg/dL)

**Mets-IR** calculation formula: Ln [(2 × FBG (mg/dL)) + TG (mg/dL)] × BMI (kg/m^2^))/(Ln [HDL-C (mg/dL)]).

### Assessment of covariates

Anthropometric measurements, behavior-related health factors, and sociodemographic data were taken into account in the analysis. Age, gender, location (urban, rural), education (primary school and below, high school, college and above), and marital status (married, never married, separated, widowed) are among the demographic factors examined. For behavioral health, we looked at drinking patterns (never, less than once a month, more than once a month), sleep time, and smoking behaviors (never, former smokers, current smokers). These facts came from self-reported records that were collected by skilled interviewers.

### Statistical analysis

We analyzed the baseline characteristics of our non-normally distributed data by calculating the median and quartiles (Q1-Q3). For categorical variables, we presented them as percentages. To stratify the data based on Mets-IR quartiles and the presence or absence of incident CVD, we used t-tests, Mann-Whitney U-tests, or Chi-square tests, as appropriate. Three logistic regression models were employed to calculate the odds ratios (ORs) and 95% confidence intervals (CIs) for incident CVD, heart disease, and stroke, using Mets-IR as a continuous (per IQR increase) or categorical (quartiles) variable. In Model 1, we accessed the crude association between METS and incident CVD. In Model 2, we added adjustments for gender, educational level, location, and marital status. Model 3 further adjusted for health behaviors, such as smoking status, alcohol consumption, and sleep duration. We performed restrictive cubic spline analyses to explore potential linear associations at specific percentiles of Mets-IR, and also visualized dose-response associations between Mets-IR and incident stroke. We performed separate interaction analyses to assess the moderating effects of sociodemographic characteristics and health-related behaviors on the association between Mets-IR and incident CVD. These models included multiplicative interaction terms, and likelihood ratio tests were conducted to assess interaction effects.

We also performed mediation analyses using the R package “mediation” to test the involvement of LDL-C in the association between Mets-IR and incident CVD/stroke/heart disease. We used bootstrapped procedures from 1000 bootstrap samples to test the importance of indirect effects. Finally, to see if Mets-IR had any additional predictive value above established clinical risk variables, we tried to fit it into a logistic regression model. We used C-statistics, continuous net reclassification improvement (NRI), and integrated discrimination improvement (IDI) for comparison purposes.

We performed all statistical analyses using R 4.1. To analyze the restricted cubic splines, we used the ‘ANOVA’ function in the rms R package. We considered statistical significance at a two-tailed P-value < 0.05.

## Result

### Characteristics of the study population according to CVD status

This study involved 4,540 participants, with a median age of 57, consisting of 2,245 males and 2,295 females. After a 7-year follow-up period from 2011 to 2018, 494 individuals (10.88%) were found to have developed cardiovascular disease (CVD), with 322 (7.09%) experiencing heart disease and 203 (4.47%) having a stroke.

**Table 1** presents the baseline characteristics of the participants at the baseline of the study, categorized by their development of CVD. The results indicate that participants who developed CVD had significantly higher baseline age and triglyceride levels, as well as lower sleep time than those who did not develop CVD. Additionally, male participants had a higher incidence of CVD (*P*=0.03). Furthermore, the result discovered that people who acquired CVD had much higher levels of Mets-IR than those who did not develop CVD.

**Table 1:** Baseline characteristics of study population by CVD status at follow-up.

	Total (*n* = 4540)	Non-CVD (*n* = 4046)	CVD (*n* = 494)	*P*
Age	57 (51, 64)	57 (51, 64)	60 (54, 66)	< 0.01
Gender				0.03
Female	2245 (49.45)	1977 (48.86)	268 (54.25)	
Male	2295 (50.55)	2069 (51.14)	226 (45.75)	
Marital				0.73
Married	4078 (89.82)	3637 (89.89)	441 (89.27)	
Non-Married	462 (10.18)	409 (10.11)	53 (10.73)	
Education				0.58
Elementary school and below	3175 (69.93)	2821 (69.72)	354 (71.66)	
High school	926 (20.4)	834 (20.61)	92 (18.62)	
College and higher	439 (9.67)	391 (9.66)	48 (9.72)	
Location				0.85
Urban	4288 (94.45)	3820 (94.41)	468 (94.74)	
Rural	252 (5.55)	226 (5.59)	26 (5.26)	
Smoking				0.11
Never	2656 (58.5)	2359 (58.3)	297 (60.12)	
Former smoker	326 (7.18)	282 (6.97)	44 (8.91)	
Current smoker	1558 (34.32)	1405 (34.73)	153 (30.97)	
Drinking				0.44
None of these	2876 (63.35)	2551 (63.05)	325 (65.79)	
Drink but less than once a month	380 (8.37)	344 (8.5)	36 (7.29)	
Drink more than once a month	1284 (28.28)	1151 (28.45)	133 (26.92)	
Sleep time	7 (5, 8)	7 (5, 8)	6 (5, 8)	< 0.01
Glucose (mg/dl)	100.26 (93.06, 108.9)	100.08 (92.88, 108.72)	101.34 (94.14, 110.47)	0.06
TG (mg/dl)	89.39 (67.26, 118.59)	88.5 (66.38, 118.59)	94.25 (72.57, 120.36)	< 0.01
HDL-C (mg/dl)	53.35 (44.85, 63.02)	53.35 (44.85, 63.02)	52.77 (44.07, 62.63)	0.45
LDL-C (mg/dl)	113.66 (94.72, 134.54)	113.27 (94.43, 134.15)	115.98 (95.88, 138.79)	0.06
Developed heart- disease				< 0.01
No	4218 (92.91)	4046 (100)	172 (34.82)	
Yes	322 (7.09)	0 (0)	322 (65.18)	
Developed stroke				< 0.01
No	4337 (95.53)	4046 (100)	291 (58.91)	
Yes	203 (4.47)	0 (0)	203 (41.09)	
Mets-IR	31.89 (28.53, 35.78)	31.8 (28.43, 35.67)	32.69 (29.33, 36.8)	< 0.01

p-Values were calculated from chi-square tests (categorical variables) or rank-sum tests (continuous variables without normal distribution), or t-tests (continuous variables with normaldistribution).

CVD, Cardiovascular disease; METS-IR, Metabolic Score for Insulin Resistance; TG, Triglyceride; HDL-C, High-density lipoprotein cholesterol; LDL-C, Low-density lipoprotein cholesterol.

### Characteristics of the study population according to quartiles of Mets-IR

**Table 2** presents the baseline characteristics of the study subjects, stratified into quartile groups based on their Mets-IR levels. Notably, as individuals’ Mets-IR levels rose, we observed a steadily rising incidence of new cardiovascular disease (CVD), with rates of 7.31%, 10.75%, 11.45%, and 14.01%, respectively. This trend was consistent for both heart disease and stroke, and the differences were statistically significant (*P*<0.01). Furthermore, we found significant differences in several baseline characteristics among the study participants, including gender, marital status, smoking status, blood glucose, TG, HDL-C, and LDL-C levels (all *P*-values < 0.05).

**Table 2:** Baseline characteristic of the study population according to Mets-IR.

Variables	Total (*n* = 4540)	Q1 (*n* = 1135)	Q2 (*n* = 1135)	Q3 (*n* = 1135)	Q4 (*n* = 1135)	*P*
Age	57 (51, 64)	55 (49, 61)	57 (51, 62)	57 (51, 63)	61 (54, 68)	< 0.01
Gender						< 0.01
Female	2245 (49.45)	564 (49.69)	592 (52.16)	590 (51.98)	499 (43.96)	
Male	2295 (50.55)	571 (50.31)	543 (47.84)	545 (48.02)	636 (56.04)	
Marital						< 0.01
Married	4078 (89.82)	1042 (91.81)	1003 (88.37)	1036 (91.28)	997 (87.84)	
Non-Married	462 (10.18)	93 (8.19)	132 (11.63)	99 (8.72)	138 (12.16)	
Education						0.32
Elementary school and below	3175 (69.93)	767 (67.58)	809 (71.28)	795 (70.04)	804 (70.84)	
High school	926 (20.4)	249 (21.94)	211 (18.59)	240 (21.15)	226 (19.91)	
College and higher	439 (9.67)	119 (10.48)	115 (10.13)	100 (8.81)	105 (9.25)	
Location						0.39
Urban	4288 (94.45)	1081 (95.24)	1075 (94.71)	1063 (93.66)	1069 (94.19)	
Rural	252 (5.55)	54 (4.76)	60 (5.29)	72 (6.34)	66 (5.81)	
Smoking						< 0.01
Never	2656 (58.5)	630 (55.51)	672 (59.21)	693 (61.06)	661 (58.24)	
Former smoker	326 (7.18)	65 (5.73)	64 (5.64)	81 (7.14)	116 (10.22)	
Current smoker	1558 (34.32)	440 (38.77)	399 (35.15)	361 (31.81)	358 (31.54)	
Drinking						0.09
None of these	2876 (63.35)	714 (62.91)	711 (62.64)	719 (63.35)	732 (64.49)	
Drink but less than once a month	380 (8.37)	108 (9.52)	86 (7.58)	110 (9.69)	76 (6.7)	
Drink more than once a month	1284 (28.28)	313 (27.58)	338 (29.78)	306 (26.96)	327 (28.81)	
Sleep time	7 (5, 8)	7 (5, 8)	6 (5, 8)	7 (5, 8)	7 (5, 8)	0.45
Glucose (mg/dl)	100.26 (93.06, 108.9)	98.46 (91.26, 106.83)	99.54 (92.7, 107.82)	100.98 (93.78, 109.62)	101.88(95.04,111.24)	< 0.01
TG (mg/dl)	89.39 (67.26, 118.59)	79.65 (61.95, 111.51)	88.5 (65.49, 122.13)	92.93 (70.36, 123.01)	95.58 (74.34, 115.93)	< 0.01
HDL-C (mg/dl)	53.35 (44.85, 63.02)	56.44 (47.17, 67.27)	55.28 (47.17, 65.34)	52.19 (44.46, 62.24)	48.71 (41.37, 57.99)	< 0.01
LDL-C (mg/dl)	113.66 (94.72, 134.54)	110.18 (90.27, 129.7)	112.11 (91.82, 132.22)	114.82 (95.68, 136.86)	118.3 (99.94, 138.79)	< 0.01
Developed heart- disease						0.01
No	4218 (92.91)	1077 (94.89)	1053 (92.78)	1050 (92.51)	1038 (91.45)	
Yes	322 (7.09)	58 (5.11)	82 (7.22)	85 (7.49)	97 (8.55)	
Developed stroke						< 0.01
No	4337 (95.53)	1108 (97.62)	1091 (96.12)	1080 (95.15)	1058 (93.22)	
Yes	203 (4.47)	27 (2.38)	44 (3.88)	55 (4.85)	77 (6.78)	
Developed CVD						< 0.01
No	4046 (89.12)	1052 (92.69)	1013 (89.25)	1005 (88.55)	976 (85.99)	
Yes	494 (10.88)	83 (7.31)	122 (10.75)	130 (11.45)	159 (14.01)	
Mets-IR	31.89 (28.53, 35.78)	27.85 (25.46, 30.19)	30.52 (28.14, 33.23)	33.2 (30.76, 35.95)	37.1 (33.75, 40.53)	< 0.01

p-Values were calculated from chi-square tests (categorical variables) or rank-sum tests (continuous variables without normal distribution), or anova (continuous variables with normal distribution).

CVD, Cardiovascular disease; METS-IR, Metabolic Score for Insulin Resistance; TG, Triglyceride; HDL-C, High-density lipoprotein cholesterol; LDL-C, Low-density lipoprotein cholesterol.

### Association between baseline Mets-IR with incident CVD/Stroke/Heart- disease

**Table 3** presents the association between Mets-IR and the risk of developing new-onset CVD, stroke, and heart disease. In the univariate model, regarding the outcome of development to CVD, the ORs were 1.09(95%CI 0.82-1.44), 1.24(95%CI 0.94-1.63) and 1.53(95% CI 1.18-2.00), respectively for the Q2, Q3, and Q4 groups, compared with the Q1 group of Mets-IR. When adjusted for different covariates combinations, the ORs for Q3 groups were significantly evaluated to1.39(95CI%=1.05,1.84), which was similar with the ORs of 1.36(95%CI 1.03-1.80) in Model 2. In fully adjusted model, Compared to the first quartile (Q1) of Mets-IR, the third (Q3) and fourth (Q4) quartiles were associated with a higher risk of new-onset CVD(OR=1.39, 95%CI=1.05-1.84, and OR=1.81, 95%CI=1.37-2.38, respectively). However, this increased risk was not observed for the second quartile (Q2)(OR=1.17, 95% CI=0.88-1.56). The results of heart disease were similar to CVD, compared to the Q1 quartile of Mets-IR, the Q4 quartile had a 55% higher risk of new-onset heart disease(OR=1.55, 95%CI=1.12-2.16), however, this increased risk was not observed for the Q2 and Q3 quartiles (OR=1.00, 95%CI=0.71-1.41, and OR=1.17, 95%CI=0.83-1.63, respectively). Furthermore, even after accounting for potential confounding factors in Model 3, the cumulative incidence of stroke exhibited a significant upward trend with increasing Mets-IR levels (*P* for trend <0.001).

**Table 3:** Associations between baseline Mets-IR with follow-up incident CVD/Stroke/Heart- disease.

	Model 1	*P*	Model 2	*P*	Model 3	*P*
CVD						
Mets-IR Per IQR	1.26 (1.11,1.43)	<0.001	1.35 (1.19,1.53)	<0.001	1.38 (1.21,1.56)	<0.001
Q1	ref		ref		ref	
Q2	1.09 (0.82,1.44)	0.567	1.16 (0.87,1.54)	0.311	1.17 (0.88,1.56)	0.279
Q3	1.24 (0.94,1.63)	0.126	1.36 (1.03,1.80)	0.030	1.39 (1.05,1.84)	0.021
Q4	1.53 (1.18,2.00)	0.002	1.75 (1.33,2.30)	<0.001	1.81 (1.37,2.38)	<0.001
*P* for trend	<0.001		<0.001		<0.001	
Heart- disease						
Mets-IR Per IQR	1.22 (1.05,1.41)	0.011	1.26 (1.08,1.47)	<0.001	1.29 (1.10,1.50)	<0.001
Q1	ref		ref		ref	
Q2	0.96 (0.68,1.34)	0.796	0.99 (0.70,1.39)	0.950	1.00 (0.71,1.41)	0.983
Q3	1.09 (0.78,1.51)	0.614	1.13 (0.81,1.59)	0.463	1.17 (0.83,1.63)	0.373
Q4	1.41 (1.03,1.93)	0.033	1.49 (1.08,2.06)	0.015	1.55 (1.12,2.16)	0.008
*P* for trend	0.020		0.009		0.005	
Stroke						
Mets-IR Per IQR	1.34 (1.11,1.61)	0.002	1.50 (1.25,1.82)	<0.001	1.51 (1.25,1.83)	<0.001
Q1	ref		ref		ref	
Q2	1.52 (0.98,2.39)	0.062	1.70 (1.09,2.67)	0.019	1.71 (1.10,2.70)	0.018
Q3	1.62 (1.05,2.53)	0.031	1.93 (1.24,3.03)	0.004	1.95 (1.26,3.07)	0.003
Q4	1.94 (1.27,2.99)	0.002	2.46 (1.60,3.83)	<0.001	2.49 (1.61,3.90)	<0.001
*P* for trend	0.003		<0.001		<0.001	

Model 1 was crude model. Model 2 was adjusted for age, gender, education level, location, and marital status. Model 3 was furthrt for smoking status, drinking status and sleep time.

CVD, Cardiovascular disease; METS-IR, Metabolic Score for Insulin Resistance; IQR, interquartile range.

When Mets-IR was examined as a continuous variable, it was discovered that each IQR rose in Mets-IR raised the risk of developing CVD by 38% (OR=1.38; 95% CI, 1.21-1.56). Similar results were observed for new-onset stroke and heart disease, with a 51% and 29% increase in risk for each IQR increase in Mets-IR by IQR, respectively (OR=1.51, 95% CI=1.25-1.83 for stroke; OR=1.29, 95% CI=1.10-1.50 for heart disease).

**Figure 1:**
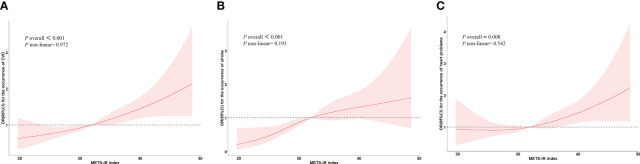
The dose-response relationships between Mets-IR and the risk of new-onset CVD, stroke, and heart disease. **(A)** Represents the relationship between the Mets-IR and the risk of CVD in restricted cubic spline. **(B)** Represents the relationship between the Mets-IR and the risk of stroke in restricted cubic spline. **(C)** Represents the relationship between the Mets-IR and the risk of heart disease in restricted cubic spline.

**Figure 1** illustrates the dose-response relationships between Mets-IR and the risk of new-onset CVD **Figure 1A**, stroke **Figure 1B**, and heart disease **Figure 1C**. The results indicate that there is a linear dose-response link between Mets-IR and the risk of new-onset CVD, stroke, and heart- disease (*P*_overall_ *<*0.05, *P*_non-linear >_0.05).

### Stratified analysis

**Figure 2:**
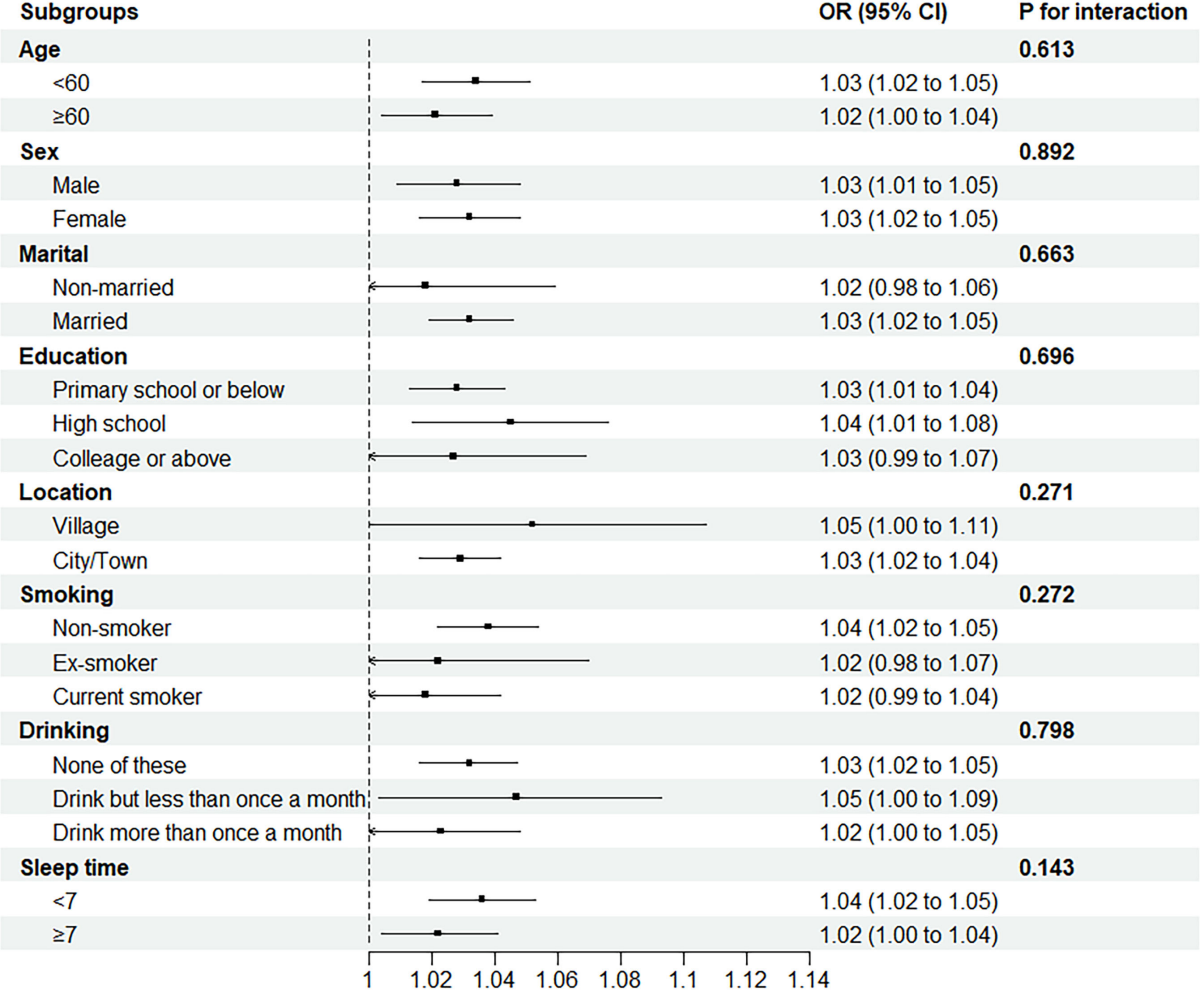
The associations between Mets-IR and incident CVD differs among subgroups.

In order to investigate if there were any differences in the associations between Mets-IR and incident CVD differs among subgroups, participants were stratified into different subgroups based on their socioeconomic characteristics and medical history **(** **Figure 2** **)**. The results showed there is no significant interactions between Mets-IR.

### Mediation analyses

Mediation analyses showed that the association between Mets-IR and incident stroke was partially mediated by LDL-C, with indirect effect estimates of 0.00002 (95% CI: 0.00001, 0.00004). In the case of the significant association between Mets-IR and incident stroke, approximately 5% (indirect effect/total effect), respectively, were mediated by LDL-C **(** **Table 4** **)**.

**Table 4:** Mediation of the Association Between Mets-IR and Incident CVD/Stroke/Heart- disease in Charls by LDL-C.

(Mets-IR)-(LDL-C)-CVD		Estimate	95%CI Lower	95%CI Upper	*P*-value
	ACME (average)	0.00001	-0.00005	0.00008	0.520
	ADE (average)	0.00120	0.00106	0.00134	<0.001 ***
	Prop. Mediated	0.01500	-0.03840	0.06840	0.520
(METS- IR)- (LDL-C)-Stroke					
	ACME (average)	0.00002	0.00001	0.00004	<0.001 ***
	ADE (average)	0.00041	0.00027	0.00054	<0.001 ***
	Prop. Mediated	0.05010	0.02400	0.07620	<0.001 ***
(METS- IR)- (LDL-C)-(Heart- disease)					
	ACME (average)	0.00000	-0.00005	0.00004	0.960
	ADE(average)	0.00083	0.00069	0.00096	<0.001***
	Prop. Mediated	0.00026	-0.05980	0.06032	0.960

CI, confidence interval; Mets-IR, Metabolic score for insulin resistance; CVD, Cardiovascular disease; LDL-C, Low-density lipoprotein cholesterol; ADE, Average Direct Effect; ACME, Average Causal Mediation Effect. *** P<0.001.

### Incremental predictive value of METS- IR

In **Table 5**, it was observed that the effect of continuous NRI and IDI on predicting CVD was significantly improved after adding Mets-IR to the basic model, with a respective increase of 0.175(*P*<0.001) and 0.006 (*P*<0.001) (**Table 5**). Furthermore, adding Mets-IR to the basic model was found to enhance the prediction of total stroke incidence, as evidenced by a noticeable increase in the C-statistic (from 0.619 to 0.646, *P*=0.031), continuous NRI (0.244, *P*<0.001) and IDI (0.004, *P*<0.001). Notably, the addition of Mets-IR also significantly improved the continuous NRI and IDI for predicting heart- disease, with an increase of 0.151(*P*=0.009) and 0.0027(*P*=0.003), respectively.

**Table 5:** Incremental predictive value of METS- IR.

	C statistic Estimate (95% CI)	*P*-value	NRI [continuous) Estimate (95% CI)	*P*-value	IDI Estimate (95% CI)	*P*-value
CVD						
Basic model	0.599 (0.573,0.626)		ref		ref	
Basic model + Mets-IR	0.615 (0.589,0.641)	0.057	0.175 (0.082,0.268)	<0.001	0.006 (0.003,0.008)	<0.001
Stroke						
Basic model	0.619 (0.579,0.659)		ref		ref	
Basic model + Mets-IR	0.646 (0.608,0.683)	0.031	0.244 (0.104,0.385)	<0.001	0.004 (0.002,0.007)	<0.001
Heart- disease						
Basic model	0.619 (0.586,0.651)		ref		ref	
Basic model + Mets-IR	0.624 (0.592,0.657)	0.409	0.151 (0.038,0.265)	0.009	0.0027 (0.000,0.004)	0.003

The basic model included age, gender, education level, location, marital status, smoking status, drinking status and sleep time.

METS- IR, Metabolic score for insulin resistance; CVD, Cardiovascular disease; NRI, Net Reclassification Improvement; IDI, Integrated Discrimination Improvement.

## Discussion

CVD has a significant prevalence worldwide and is one of the primary causes of death, which has a serious impact on human health. It was suggested in our research that the Mets-IR index was a significant positive correlation with the risk of new CVD/Stroke/Heart- disease, and this association was independent of age, gender, smoking, history of alcohol consumption, and sleep duration. In comparison to the first quartile of Mets-IR (Q1), blood sugar and cholesterol levels were considerably higher in Mets-IR (Q4). In addition, the dose-response curve’s findings demonstrated a linear relationship between Mets-IR and CVD/Stroke/Heart- disease, with increasing risk of new CVD/Stroke/Heart- disease with increasing Mets-IR when Mets-IR exceeded a certain threshold. Moreover, LDL-C mediates the relationship between Mets-IR and stroke onset. Interestingly, combining Mets-IR parameters with the basic model significantly improves the efficacy of the basic model in predicting the occurrence of CVD/Stroke/Heart- disease.

Insulin resistance refers to the reduced responsiveness of the body to the physiological effects of insulin. Its formation mechanism is very complex, mostly brought about by a confluence of genetic predisposition and unfavorable environmental conditions (17). Numerous investigations have demonstrated a tight connection between insulin and the development of type 2 diabetes, meanwhile, insulin resistance has adverse effects on the progression of atherosclerosis and CVD (18). A possible mechanism is that insulin resistance is often accompanied by vascular endothelial cell dysfunction, activation of the coagulation system, and increased levels of fibrinogen activation inhibitor-1 in plasma, which leads to lipid deposition and thrombosis, causing coronary heart disease (19). In addition, insulin resistance causes disorders of glucolipid metabolism and induces chronic inflammation, which also increases the risk of cardiovascular disease.

The well-known “gold standard” indicator for accessing insulin resistance has been the hyperinsulinemic-euglycemic (HIEG) clamp test (20). However, it is time-consuming, expensive and not suitable for large-scale applications. Mets-IR is a new insulin resistance index that combines FBG, TG, HDL-C laboratory indices and BMI measure indices. The Mets-IR index has high accuracy in detecting insulin resistance as validated by the glycemic-hyperinsulinemic clamp (EHC), with an AUC of 0.84 (95% CI: 0.78-0.90) (13). Moreover, the FBG, TG, HDL-C and BMI indices are easy to obtain, and together with our findings, the Mets-IR index may be trustworthy indication to assess the prediction of CVD risk.

We discovered a trend toward a favorable connection between Mets-IR and the risk of cardiovascular disease. This outcome is consistent with earlier research. The triglyceride glucose (TyG) index is a reliable surrogate for assessing insulin sensitivity, and Baydar et al. found that TyG is associated with subclinical atherosclerosis (21). In addition, insulin resistance is significantly correlated with coronary heart disease severity. Zhao et al. (22) considered that the TyG index is positively associated with the risk of coronary heart disease (CHD) and coronary atherosclerosis severity among non-alcoholic fatty liver disease (NAFLD) patients. Unlike them, we used Mets-IR- a novel insulin resistance index, which, compared with TyG, has a higher predictive value in predicting the existence as well as the severity of coronary artery disease (16).

Several previous large studies have confirmed the strong association of Mets-IR with hypertension, coronary artery calcification, and ischemic heart disease. It was suggested that Mets-IR levels were positively linked with the prevalence of prehypertension or hypertension according to a retrospective study from Japan that included 15,453 subjects (23). Wang et al. discovered that Mets-IR may have better discriminatory power than other IR indicators in predicting the incidence of coronary artery calcification in asymptomatic adults without cardiovascular disease (CVD) (24). Mets-IR is also a reliable predictor of coronary heart disease severity with an AUC of 0.726 (95% CI 0.677-0.775) (25). A large longitudinal cohort study of Korean adults who were not diabetic found that higher Mets-IR levels were linked with an increased risk of new-onset ischemic heart disease (IHD) (26). The results mentioned above support our conclusion that MTES-IR levels are substantially correlated with the probability of developing a new CVD.

The mediating effect suggests a mediating role for LDL-C between Mets-IR and stroke development. This suggests that the influence of Mets-IR on stroke is partly achieved through changes in LDL-C levels. The potential mechanisms are as follows: Insulin resistance affects cholesterol synthesis and metabolism in adipose tissue and the liver, leading to increased synthesis of LDL-C and hindered LDL-C degradation (27). Elevated LDL-C levels may contribute to the risk of stroke by promoting atherosclerosis and thrombus formation, resulting in vascular obstruction and cerebral ischemia (28, 29).

Finally, we added Mets-IR to the basic model (model 3) to observe the change in model efficacy, and we found that the model predictive value increased significantly with the addition of Mets-IR parameters. This finding further substantiates our claim that METS-IR serves as a reliable predictor for the risk of new-onset CVD, stroke, and heart disease.

Our study has certain advantages. First, to the best of our knowledge, it is the first time to carry out a prospective investigation to explore the relationship between Mets-IR and the risk of new CVD/Stroke/Heart- disease. Our findings further confirm that insulin resistance is strongly associated with the occurrence of cardiovascular and cerebrovascular events with a cumulative risk of dose. Secondly, this is a nationwide study based on a large and representative sample with high feasibility of results. Finally, the design of the prospective cohort study allowed the results to elucidate the causal relationship between insulin resistance and cardiovascular events to a certain extent, providing a reference for reducing the occurrence of cardiovascular and cerebrovascular events.

However, our study also has some limitations. First, CVD was based on self-report, and although the method is relatively reliable (30), it cannot completely avoid the possibility of misreporting. Second, although we corrected for a variety of factors, we were unable to correct for genetic factors, diet, and other factors due to data limitations. Third, the participants included in this investigation were all middle-aged as well as older Chinese population aged ≥45 years; thus, the applicability of our findings to people from other countries, or ≤45 years old, remains to be verified. In order to establish a solid scientific foundation for the prevention of cardiovascular diseases, additional prospective cohort studies are required to examine the association between the Mets-IR index and the risk of CVD, stroke, and heart disease.

## Conclusion

In conclusion, we considered that Mets-IR was associated with the risk of CVD/Stroke/Heart- disease, and LDL-C played a mediating role between the above relationships. Improving insulin sensitivity and regulating lipids may be important and effective measures to prevent cardiovascular events. Our study has considerable public health implications for the prevention of cardiovascular events.

## Data availability statement

Publicly available datasets were analyzed in this study. This data can be found here: http://charls.pku.edu.cn.

## Author contributions

TQ wrote the main manuscript text. XS and PS analyzed data. YF and YD drew the tables and figures in the manuscript. GZ guided the direction of the manuscript and supervised the completion of this manuscript. All authors contributed to the article and approved the submitted version.
